# Perivascular space enlargement accelerates in ageing and Alzheimer’s disease pathology: evidence from a three-year longitudinal multicentre study

**DOI:** 10.1186/s13195-024-01603-8

**Published:** 2024-10-31

**Authors:** Inga Menze, Jose Bernal, Pinar Kaya, Çağla Aki, Malte Pfister, Jonas Geisendörfer, Renat Yakupov, Roberto Duarte Coello, Maria d. C. Valdés-Hernández, Michael T. Heneka, Frederic Brosseron, Matthias C. Schmid, Wenzel Glanz, Enise I. Incesoy, Michaela Butryn, Ayda Rostamzadeh, Dix Meiberth, Oliver Peters, Lukas Preis, Dominik Lammerding, Daria Gref, Josef Priller, Eike J. Spruth, Slawek Altenstein, Andrea Lohse, Stefan Hetzer, Anja Schneider, Klaus Fliessbach, Okka Kimmich, Ina R. Vogt, Jens Wiltfang, Claudia Bartels, Björn H. Schott, Niels Hansen, Peter Dechent, Katharina Buerger, Daniel Janowitz, Robert Perneczky, Boris-Stephan Rauchmann, Stefan Teipel, Ingo Kilimann, Doreen Goerss, Christoph Laske, Matthias H. Munk, Carolin Sanzenbacher, Petra Hinderer, Klaus Scheffler, Annika Spottke, Nina Roy-Kluth, Falk Lüsebrink, Katja Neumann, Joanna Wardlaw, Frank Jessen, Stefanie Schreiber, Emrah Düzel, Gabriel Ziegler

**Affiliations:** 1 German Centre for Neurodegenerative Diseases (DZNE), Leipziger Str. 44, Magdeburg, 39120 Germany; 2 Institute of Cognitive Neurology and Dementia Research, Otto-Von-Guericke University Magdeburg, Leipziger Str. 44, Magdeburg, 39120 Germany; 3 Centre for Clinical Brain Sciences, The University of Edinburgh, 49 Little France Crescent, Edinburgh, EH16 4SB UK; 4 UK Dementia Research Institute Centre at the University of Edinburgh, Edinburgh Bioquarter, 49 Little France Crescent, Edinburgh Bioquarter, Edinburgh, EH16 4SB UK; 5 Department of Neurology, University Hospital Magdeburg, Leipziger Str. 44, 39120 Magdeburg, Germany; 6 Luxembourg Centre for Systems Biomedicine (LCSB), University of Luxembourg, Belvaux, 6 Avenue du Swing 4367 , Esch-Belval, Luxembourg; 7 German Centre for Neurodegenerative Diseases (DZNE), Venusberg-Campus 1, Bonn, 53127 Germany; 8 Institute for Medical Biometry, Informatics and Epidemiology, University Hospital Bonn, Venusberg-Campus 1, Bonn, 53127 Germany; 9 Department of Psychiatry and Psychotherapy, University Hospital Magdeburg, Leipziger Str. 44, Magdeburg, 39120 Germany; 10 Department of Psychiatry, Medical Faculty, University of Cologne, Kerpener Strasse 62, Cologne, 50924 Germany; 11 German Centre for Neurodegenerative Diseases (DZNE), Charitéplatz 1, Berlin, 10117 Germany; 12 Institute of Psychiatry and Psychotherapy, Freie Universität Berlin and Humboldt-Universität zu Berlin, Hindenburgdamm 30, Berlin, 12203 Germany; 13 Department of Psychiatry and Psychotherapy, Charité, Charitéplatz 1, Berlin, 10117 Germany; 14 School of Medicine, Department of Psychiatry and Psychotherapy, Technical University of Munich, Ismaninger Str. 22, Munich, 81675 Germany; 15 Berlin Center for Advanced Neuroimaging, Charité, Charitéplatz 1, Berlin, 10117 Germany; 16 Department of Neurodegenerative Disease and Geriatric Psychiatry/Psychiatry, University of Bonn Medical Center, Venusberg-Campus 1, Bonn, 53127 Germany; 17 German Centre for Neurodegenerative Diseases (DZNE), Von-Siebold-Str. 3a, 37075 Goettingen, Germany; 18 Department of Psychiatry and Psychotherapy, University Medical Center Goettingen, Von-Siebold-Str. 5, Goettingen, 37075 Germany; 19 Neurosciences and Signaling Group, Institute of Biomedicine (iBiMED), Department of Medical Sciences, University of Aveiro, Campus Universitário de Santiago, Aveiro, 3810-193 Portugal; 20 Leibniz Institute for Neurobiology, Brenneckestraße 6, Magdeburg, 39118 Germany; 21 Department of Cognitive Neurology, MR-Research in Neurosciences, Georg-August-University Goettingen, Robert-Koch-Straße 40, Göttingen, 37075 Germany; 22 German Centre for Neurodegenerative Diseases (DZNE), Feodor-Lynen-Strasse 17, Munich, 81377 Germany; 23 Institute for Stroke and Dementia Research (ISD), University Hospital, LMU Munich, Feodor-Lynen-Strasse 17, Munich, 81377 Germany; 24 Department of Psychiatry and Psychotherapy, University Hospital, LMU Munich, Nußbaumstraße 7, Munich, München 80336 Germany; 25 Munich Cluster for Systems Neurology (SyNergy), Feodor-Lynen-Str. 17, Munich, 81377 Germany; 26 Ageing Epidemiology Research Unit (AGE), School of Public Health, Imperial College London, Charing Cross Hospital, St Dunstan’s Road, London, W6 8RP UK; 27 Sheffield Institute for Translational Neuroscience (SITraN), University of Sheffield, 385a Glossop Rd, Sheffield, Broomhall, Sheffield, S10 2HQ UK; 28 Department of Neuroradiology, University Hospital LMU, Marchioninistr. 15, Munich, 81377 Germany; 29 German Centre for Neurodegenerative Diseases (DZNE), Gehlsheimer Straße 20, Rostock, 18147 Germany; 30 Department of Psychosomatic Medicine, Rostock University Medical Center, Gehlsheimer Straße 20, Rostock, 18147 Germany; 31 German Centre for Neurodegenerative Diseases (DZNE), Otfried-Müller-Straße 23, Tübingen, 72076 Germany; 32 Section for Dementia Research, Hertie Institute for Clinical Brain Research and Department of Psychiatry and Psychotherapy, University of Tübingen, Osianderstraße 24, Tübingen, 72076 Germany; 33 Department of Psychiatry and Psychotherapy, University of Tübingen, Osianderstraße 24, Tübingen, 72076 Germany; 34 Department for Biomedical Magnetic Resonance, University of Tübingen, Otfried-Müller-Straße 51, Tübingen, 72076 Germany; 35 Department of Neurology, University of Bonn, Venusberg-Campus 1, Bonn, 53127 Germany; 36 Excellence Cluster On Cellular Stress Responses in Aging-Associated Diseases (CECAD), University of Cologne, Joseph-Stelzmann-Straße 26, Cologne, 50931 Germany

**Keywords:** Enlarged perivascular spaces, Virchow–Robin spaces, Alzheimer’s disease, Alzheimer’s pathology, Longitudinal analysis, Multicentre study

## Abstract

**Background:**

Perivascular space (PVS) enlargement in ageing and Alzheimer’s disease (AD) and the drivers of such a structural change in humans require longitudinal investigation. Elucidating the effects of demographic factors, hypertension, cerebrovascular dysfunction, and AD pathology on PVS dynamics could inform the role of PVS in brain health function as well as the complex pathophysiology of AD.

**Methods:**

We studied PVS in centrum semiovale (CSO) and basal ganglia (BG) computationally over three to four annual visits in 503 participants (255 females; mean_age_ = 70.78 ± 5.78) of the ongoing observational multicentre “DZNE Longitudinal Cognitive Impairment and Dementia Study” (DELCODE) cohort. We analysed data from subjects who were cognitively unimpaired (*n* = 401), had amnestic mild cognitive impairment (*n* = 71), or had AD (*n* = 31). We used linear mixed-effects modelling to test for changes of PVS volumes in relation to cross-sectional and longitudinal age, as well as sex, years of education, hypertension, white matter hyperintensities, AD diagnosis, and cerebrospinal-fluid-derived amyloid (A) and tau (T) status (available for 46.71%; A-T-/A + T-/A + T + *n* = 143/48/39).

**Results:**

PVS volumes increased significantly over follow-ups (CSO: *B* = 0.03 [0.02, 0.05], *p* < 0.001; BG: *B* = 0.05 [0.03, 0.07], *p* < 0.001). PVS enlargement rates varied substantially across subjects and depended on the participant’s age, white matter hyperintensities volumes, and amyloid and tau status. PVS volumes were higher across elderly participants, regardless of region of interest (CSO: *B* = 0.12 [0.02, 0.21], *p* = 0.017; BG: *B* = 0.19 [0.09, 0.28], *p* < 0.001). Faster BG-PVS enlargement related to lower baseline white matter hyperintensities volumes (*ρ*_*spearman*_ = -0.17, *p*_*FDR*_ = 0.001) and was more pronounced in individuals who presented with combined amyloid and tau positivity versus negativity (A + T +  > A-T-, *p*_*FDR*_ = 0.004) or who were amyloid positive but tau negative (A + T +  > A + T-, *p*_*FDR*_ = 0.07). CSO-PVS volumes increased at a faster rate with amyloid positivity as compared to amyloid negativity (A + T-/A + T +  > A-T-, *p*_*FDR*_ = 0.021).

**Conclusion:**

Our longitudinal evidence supports the relevance of PVS enlargement in presumably healthy ageing as well as in AD pathology. We further discuss the region-specific involvement of white matter hyperintensities and neurotoxic waste accumulation in PVS enlargement and the possibility of additional factors contributing to PVS progression. A comprehensive understanding of PVS dynamics could facilitate the understanding of pathological cascades and might inform targeted treatment strategies.

**Trial registration:**

German Clinical Trials Register DRKS00007966. Registered 04.05.2015 – retrospectively registered, https://drks.de/search/en/trial/DRKS00007966.

**Supplementary Information:**

The online version contains supplementary material available at 10.1186/s13195-024-01603-8.

## Background

Perivascular spaces (PVS) are millimetre-sized, fluid-filled compartments surrounding small perforating cerebral vessels [1, 2]. PVS can become large enough to be visible in magnetic resonance imaging (MRI) in humans—a phenomenon that is increasingly discussed in the context of healthy and pathological ageing. PVS visibility has generally been found associated with ageing [3–8] and large cross-sectional lifespan studies suggest that it may follow a second-order polynomial pattern throughout life, with both periods of acceleration and saturation [7, 8]. Arterial hypertension [4, 9, 10] has also been found to contribute to PVS visibility, particularly at the level of the lenticular nuclei, where small, penetrating end arteries branch off from larger, high-pressure vessels such as the middle cerebral artery [11]. Other factors including structural (e.g. capillary tortuosity; Fig. 3B in [12]) or functional (e.g. blood flow) cerebral small vessel alterations [12], and systemic- or neuroinflammation [13, 14] have also been linked to increased PVS visibility.

The likely multifactorial aetiology of PVS enlargement in humans remains, so far, elusive [12]. The hypothesised role of PVS in the removal of metabolic and neurotoxic waste products, including Amyloid-β (Aβ) and tau [15–17]—key proteins in the pathogenesis of Alzheimer’s disease (AD)—proposes that PVS enlargement reflects compromised PVS function and, by extension, potentially dysregulated glymphatic fluid exchange and clearance (for review see [2, 15, 18, 19]). In line with the clearance hypothesis, PVS burden has been found associated with elevated levels of Aβ and tau [20, 21], vascular Aβ deposition in cerebral amyloid angiopathy [22–24], as well as with clinical diagnosis of mild cognitive impairment (MCI) [25] and AD [26, 27]. Since markers of cerebral small vessel disease (CSVD) are increasingly discussed as potentially aggravating factors in AD pathology [28, 29], PVS alterations could indicate an early link between CSVD and AD.

While the aforementioned evidence and deductions are compelling, some studies did not observe clear associations of AD diagnosis or pathology with PVS burden [30, 31], casting doubt on the involvement of PVS alterations in AD and the validity of the clearance hypothesis. Longitudinal studies that could establish specific factors that mechanistically drive PVS enlargement are scarce due to the persistent methodological challenges to quantify PVS reliably and computationally using repeated measures [32]. A few prospective studies in healthy ageing suggest that PVS counts [5, 33] and volumes [6] increase over time, with their baseline load contributing to both the progression of PVS and other markers of CSVD, especially white matter hyperintensities (WMH) [5, 6]. As such, numerous cross-sectional studies support the positive association between PVS and WMH [2] and a few even provide evidence of WMH developing in the proximity of PVS [34]. Other studies nonetheless found only partial or no evidence for PVS frequency increases over time in healthy ageing [3] or CSVD [35]. Taken together, these findings point to the relevance of further research into conditions that contribute to the presence and dynamical changes of PVS, particularly in the context of a complex multifactorial neurodegenerative disease such as AD.

In this study, we quantified and monitored PVS volumes in a large longitudinal multicentre study (503 subjects along AD syndromal cognitive stages; 4 annual time points, 1791 multimodal structural MRI scans) using a multimodal segmentation approach. Leveraging longitudinal modelling, we sought to characterise dynamical PVS changes in presumably healthy ageing and across AD syndromal cognitive stages. We anticipated (a) subjects with a history of hypertension would exhibit increased PVS volumes at baseline and more pronounced volume increases over time than normotensive individuals, (b) WMH of presumed vascular origin would relate to PVS dynamics, and (c) diagnosis (MCI and AD versus controls) and presence of AD biomarkers (amyloid and/or tau positivity).

## Methods

### Study design and participants

We used baseline and annual follow-up data for up to 36 months from cognitively unimpaired participants (CU), as well as patients with mild cognitive impairment (MCI) and AD, enrolled in DELCODE (DZNE Longitudinal Cognitive Impairment and Dementia Study [36]; see Supplementary Fig. 1, Additional File 1)—an observational multicentre study from the German Centre for Neurodegenerative Diseases (DZNE).

The definition of the CU group and the allocation to diagnostic groups in DELCODE followed the existing research criteria and are described in detail in [36], along with the additional exclusion and inclusion criteria. All participants gave written informed consent in accordance with the Declaration of Helsinki prior joining the study. DELCODE is retrospectively registered at the German Clinical Trials Register (DRKS00007966, 04/05/2015).

#### Hypertension

We categorised subjects into normotensive and hypertensive according to their ICD-10 diagnosis, as described in [37]. This information was available for 501 subjects (99.60%). As most subjects with hypertension (*n* = 273, 54.49%) had been prescribed antihypertensive medication (*n* = 262, 95.97%), we refer to this group as treated hypertensive subjects.

#### CSF biomarker assessment and AD biomarker profiles

We classified individuals based on their CSF-derived amyloid- (A) and tau-positivity status (T) into A-T-, A-T + , A + T-, and A + T + [28] (*n* = 143, 5, 48, 39 respectively). CSF biomarker samples were obtained through lumbar puncture [36]. We used DELCODE-specific cut-offs to determine biomarker positivity (A-: Aβ42/40 > 0.08; A + : Aβ42/40 ≤ 0.08; T-: pTau181 < 73.65; T + : pTau181 ≥ 73.65; see [38] for more information).

Solely in analyses investigating PVS change rates in relation to AD biomarker profiles, we excluded the A-T + group due to its small sample size and since it is not considered to reflect AD pathological changes [28].

#### Structural magnetic resonance imaging

MRI acquisition took place at nine DZNE sites equipped with 3 T Siemens MR scanners (see Supplementary Table 1 & 2, Additional File 1 for scanner details). The DZNE imaging network oversaw operating procedures and quality assurance and assessment (iNET, Magdeburg) [36].

For *white matter and grey matter segmentation with FreeSurfer*, we used T1w MPRAGE images (full head coverage; 3D acquisition, GRAPPA factor 2, 1 mm^3^ isotropic, 256 × 256 px, 192 sagittal slices, TR/TE/TI 2500/4.33/1100 ms, FA 7°).

For *visual rating of PVS in the centrum semiovale (CSO) and basal ganglia (BG)*, we employed T2w imaging (partial head coverage; 0.5 × 0.5 × 1.5 mm, 384 × 384 px, 64 quasi-coronal slices perpendicular to hippocampal long axis, TR/TE 3500/353 ms). We also used T2w turbo spin-echo images (full head coverage; 0.8 × 0.8 × 2 mm, 240 × 320 px, 72 axial slices, TR/TE 6500/79 ms; available in *n* = 214 at baseline) in a subsample of 30 subjects to assess absolute PVS counts in the slice with the highest PVS burden.

For *computational PVS and WMH quantification*, we used T1w MPRAGE and T2w FLAIR (full head coverage; 1 mm^3^ isotropic, 256 × 256 px, 192 sagittal slices, TR/TE/TI 5000/394/1800 ms) images. We opted for T1w and FLAIR imaging for PVS assessment over T2w imaging because T2w images were either anisotropic, not available for all individuals or did not cover the entire brain (Supplementary Fig. 2, Additional File 1), thereby limiting the availability, reliability and quality of PVS quantification.

### Segmentation and quantification

#### Total intracranial volume

We estimated segmentation-based total intracranial volume (sbTIV) using Freesurfer’s samseg-based structure segmentation [39] and adjusted for it as a covariate in all models (cf. [40–42]. We did not use fractional volumes because they depend on the region of interest's volume, which can change over time, esp. in groups with pathologies such as AD. This would render interpretation more difficult, e.g. if an increase in fractional PVS volumes reflects an absolute increase in PVS volumes or a shrinkage of the region of interest (Supplementary Fig. 3 A-B, Additional File 1).

#### Segmentation of PVS and WMH of presumed vascular origin

We segmented PVS computationally in all enrolled and eligible subjects with T1w and FLAIR imaging (*n* = 871) using a thoroughly validated PVS segmentation software [43, 44].

The PVS segmentation software segmented both the Region-of-Interest (ROI) and the PVS in each of these regions. We created CSO and BG ROIs, in accordance with Potter’s scale [45], based on T1w and FLAIR images. Here, the BG ROI is defined by the internal and external capsules as well as caudate, lentiform and thalamic nuclei. CSO is defined as the remaining supratentorial white matter. Although the ROIs do not correspond to precise anatomical structures, we retained the nomenclature to align with the visual rating methods commonly employed in the field.

We segmented hyperintensities of presumed vascular origin using a hierarchical thresholding approach leveraging T1w and FLAIR imaging and used the resulting segmentations to estimate their volumes within each ROI. We retrieved subcortical hyperintensities within the BG ROI [46], WMH in the CSO ROI (CSO-WMH). We refer to both subcortical hyperintensities and CSO-WMH as “WMH of presumed vascular origin”. We restricted our PVS measurements to the normal-appearing brain parenchyma (NABP) since measuring PVS inside WMH was unreliable in T1w imaging.

Further details on the segmentation process are available in Supplementary Section 2.2.2, Additional File 1.

##### Segmentation and parameter tuning

We segmented PVS using the Frangi filter and thresholding [47, 48]. We optimised segmentation thresholds per ROI to maximise the correlation between qualitative and computational estimates of PVS burden (CSO = 2.5074 × 10^–6^, BG = 1.6854 × 10^–5^), as in [48]. Further information about the optimisation procedure can be found in Supplementary Section 2.2.2.1, Additional File 1, and evidence of the segmentation across different acquisition sites in Additional File 2. Qualitative PVS ratings were provided by two neurology residents (MP and JG) blinded to clinical data, who independently rated PVS in the CSO and BG on T2w images with partial head coverage using the Potter’s rating procedure [45]. We used connected component analysis to filter out structures that were either too large or too small (structures spanning more than 200 voxels and less than 2 voxels, respectively) [49]. We also eliminated structures that were positioned completely at white matter perimeter in order to mitigate partial volume effects [49, 50]. Finally, we estimated total PVS volumes within each ROI.

##### Clinical validation and rescan reliability of PVS

A third independent neurology resident (PK), blinded to clinical data, counted PVS in the axial slice with the highest burden separately for CSO and BG. This procedure was carried out for 30 subjects with available T2w scans with full head coverage, who were randomly drawn from the initially enrolled and eligible pool of subjects (11 females; mean_age_ = 70 ± 4.35 years). We compared their absolute counts to our estimated computational counts in the same slice and to computationally estimated PVS volumes. We used Spearman’s correlations, Lin's concordance correlation coefficient (CCC), and Bland–Altman plots for this purpose.

We also assessed the stability of computational PVS segmentation over time by correlating PVS volumes—controlled for age, sex, years of education and sbTIV—across time points.

##### Comparison to PVS segmentation in subsample with T2w scans

We segmented PVS in T2w at baseline in the subsample with available T2w full head coverage (*n* = 214) and compared those estimates against those obtained from T1w imaging. We followed the afore-noted steps of clinical validation to compare segmentation from both imaging sequences (Supplementary Fig. 6A-D, Additional File 1).

### Statistical analyses

#### Data transformation and adjustment of baseline data

Volumes of PVS and of WMH of presumed vascular origin and sbTIV were Box-Cox transformed (model with intercept only), to account for non-normality and skewness, and then z-scored. We z-scored years of education and age. We corrected baseline volumes of WMH of presumed vascular origin and PVS for linear and quadratic age effects, sex, years of education and sbTIV via residualisation.

#### Estimating PVS rates of change

We estimated group level as well as subject-specific *rates of change* in CSO- and BG-PVS volumes via linear mixed-effects (LME) modelling. Following recommendations to assess robust linear trends via LME models (cf. [51, 52]), we used data from 503 individuals who attended at least three MRI scanning sessions (1791 sets of T1w and FLAIR) to obtain reliable PVS rates of change even in light of missing data point [53]. Moreover, in line with the work of Guillaume et al. [54], we incorporated a longitudinal 'visit'—or here *time*—effect (centred intra-subject age) and a cross-sectional 'age' effect into our models. All LME models included correlated random intercepts and random slopes and accounted for linear and quadratic age effects, sex, years of education and sbTIV. We extracted subject-specific slopes from the fitted LME models that denote the individual-level rate of change in PVS volumes over time (follow-ups), that moreover accounted volumes of WMH of presumed vascular origin volumes.

We visually screened residual plots and normality via Q-Q plots to check linearity and homoscedasticity. We excluded outliers (standard residual greater than 3 SD) and influential data points exceeding a cut-off value suggested by Van der Meer and colleagues [55] (Cook’s distance > 4/n). We reported effect size estimates using beta coefficients, 95% confidence intervals and an alpha level of *p* < 0.05.

To provide a rough yet interpretable estimate of change we estimated average annual increase of PVS volumes in ml/year and change from baseline in %/year for each diagnostic group and with respect to AD biomarker profile. For each group, we computed mean PVS volumes at each time point, accounting for age, and approximated the annual rates of change with linear models.

#### Healthy ageing versus AD

We conducted two separate statistical analyses with the aim of disentangling the contributions of presumably healthy ageing alone but also in conjunction with AD pathology: one for the CU group and one in the entire cohort (i.e., CU, MCI, and AD).

In CU participants, we tested whether baseline PVS volumes and their rates of change were explained by ageing, hypertension and WMH. We examined the relationships with aging directly within the LME model. We used the Mann–Whitney-U Test to test for differences in baseline PVS volumes and PVS rates of change between treated hypertensive and normotensive cases. We used correlational analysis to determine whether baseline regional WMH volumes explained part of the variance in regional PVS volumes at baseline and PVS rates of change.

In the entire sample, we used the Kruskal–Wallis-tests and FDR-corrected post-hoc comparisons to test whether baseline PVS volumes and PVS rates of change varied across diagnostic groups and AD biomarker profile.

#### Software

We analysed data with *R* (v4.0.2) using RStudio (v1.3.1073) [56]. We modelled and diagnosed linear mixed effect models (LME) with *lme4*, *lmerTest*, *influence.ME* and *psych*. Correlations and group differences (Mann–Whitney U-tests, Kruskal Wallis tests, ANOVAs) were conducted with *rstatix.* We created figures with *ggplot2*, *ggpubr*, and *ITK-SNAP* [57].

## Results

### Clinical validation and rescan reliability of PVS

**Fig. 1:**
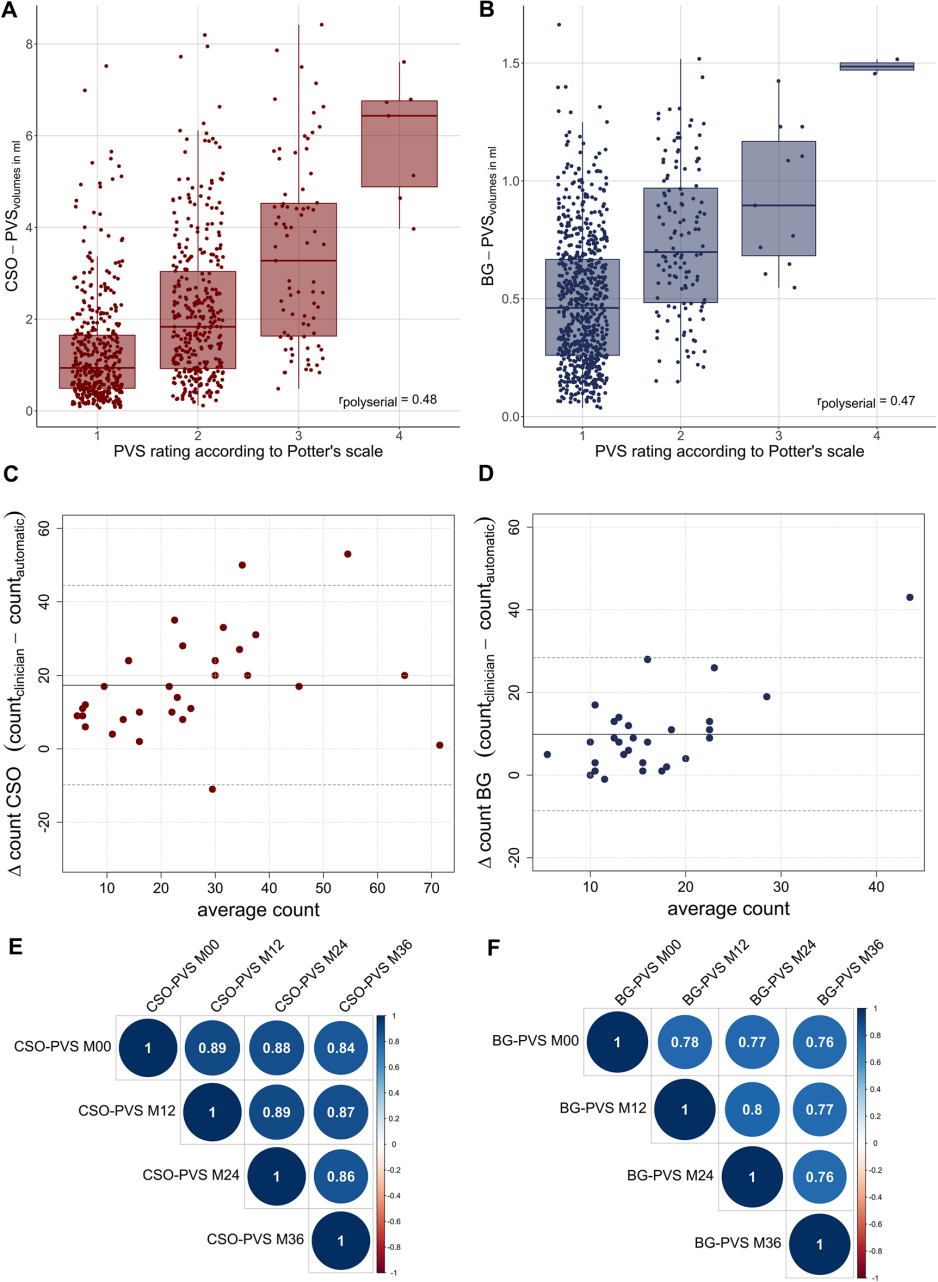
Clinical validation and reliability using repeated measures. **A-B** Moderate polyserial correlations between PVS volumes and visual scores. **C-D** Bland–Altman plot comparing manual and computational PVS counts in the axial slice with the highest PVS burden in white matter and basal ganglia in a subset of 30 random subjects. Solid lines depict the mean difference and dotted lines depict the corresponding 95%-confidence intervals. **E–F** High Pearson’s correlations of CSO- and BG-PVS volumes across four annual time points suggest measurement stability. PVS volumes were Box-Cox transformed and z-scored to account for skewness and corrected for linear and quadratic age effects, years of education, sex and total intracranial volume

First, we obtained moderate polyserial correlations between computational PVS assessments and clinical PVS ratings from T2w images with partial head coverage (CSO: *r*_*polyserial*_ = 0.48, *p* < 0.001; BG: *r*_*polyserial*_ = 0.47, *p* < 0.001; Fig. 1A, B).

In the subset of 30 randomly selected subjects with T2w full head coverage, manual counts in the slice with the highest PVS burden and computational volumes correlated strongly (CSO: *ρ*_*spearman*_ = 0.69, *p* < 0.001; BG: *ρ*_*spearman*_ = 0.50, *p* = 0.006). Associations between computationally assessed and manual counts in the same slice were strong in CSO (*ρ*_*spearman*_ = 0.76, *p* < 0.001) and moderate in BG (*ρ*_*spearman*_ = 0.38, *p* = 0.041). We noticed an underestimation of PVS counts by our computational approach (CSO: *difference*_*mean*_ = 17.33, *difference*_*SD*_ = 13.84; BG: *difference*_*mean*_ = 9.87, *difference*_*SD*_ = 9.44), which resulted in moderate Lin's concordance correlation coefficients (CSO: *CCC* = 0.49, *95%-CI* [0.28, 0.66]; BG: *CCC* = 0.23, *95%-CI* [0.06, 0.39]). In addition, the Bland–Altman plot suggested a proportional discrepancy: the more PVS there were, the fewer PVS the computational method detected (Fig. 1C, D). Given that PVS sensitivity is modality specific, such underestimation is expected.

Correlation of computational PVS segmentation across repeated measures was high for both CSO (Fig. 1E) and BG (Fig. 1F) suggesting high reliability.

Apart from the scarcity of eligible T2w-imaging, validation results of computational PVS segmentation from T1w and T2w images yielded comparable results, allowing valid interpretation and use of the PVS characterization based on T1w scans (Supplementary Fig. 6 , Additional File 1).

### Descriptive statistics and sample characteristics

In this study we quantified interindividual PVS enlargement over time in 503 DELCODE participants (Supplementary Fig. 1, Additional File 1). Detailed sample characteristics including diagnostic groups are reported in Table 1.

**Table 1:** Descriptive statistics for CSO-PVS and BG-PVS volumes (in ml) for four annual visits, stratified by clinical groups. Group-level results included subjects who had at least three scans available (*n* = 503). We removed extreme values in volumes within the whole sample for each time point to lessen the influence of outliers and corrected estimates for age at the time of the scan

		***Clinical Group***		
		**CU**	**MCI**	**AD**
	**N**	*401*	*71*	*31*
*Baseline demographics*	**Females (%)**	52.12	40.85	54.84
	**Age (years)**	70.2 ± 0.28	72.5 ± 0.69	74.3 ± 1.12
	**Education (years)**	14.9 ± 0.15	14.0 ± 0.37	12.9 ± 0.50
*Baseline volume (ml)*	**CSO-PVS**	1.91 ± 0.08	2.02 ± 0.20	1.56 ± 0.30
	**BG-PVS**	0.54 ± 0.02	0.60 ± 0.04	0.42 ± 0.06
*12-month follow-up volume (ml)*	**CSO-PVS**	2.06 ± 0.09	2.02 ± 0.21	1.81 ± 0.32
	**BG-PVS**	0.57 ± 0.02	0.59 ± 0.04	0.44 ± 0.06
*24-month follow-up volume (ml)*	**CSO-PVS**	1.99 ± 0.09	2.28 ± 0.21	1.84 ± 0.33
	**BG-PVS**	0.58 ± 0.02	0.63 ± 0.04	0.46 ± 0.06
*36-month follow-up volume (ml)*	**CSO-PVS**	2.07 ± 0.11	2.21 ± 0.26	1.66 ± 0.49
	**BG-PVS**	0.57 ± 0.02	0.62 ± 0.05	0.42 ± 0.09
*Annual change*^*a*^* of PVS (in ml/year)*	**CSO-PVS**	0.04 ± 0.03	0.08 ± 0.04	0.03 ± 0.07
	**BG-PVS**	0.01 ± 0.00	0.01 ± 0.01	0.002 ± 0.01
*Annual change*^*a*^* of PVS from baseline (in %/year)*	**CSO-PVS**	2.11	4.13	1.98
	**BG-PVS**	1.82	1.68	0.46

Annotations. Mean ± standard error;

^a^Change of PVS volumes per AD biomarker group was estimated based on linear approximations

### PVS enlargement in cognitively unimpaired individuals

**Fig. 2:**
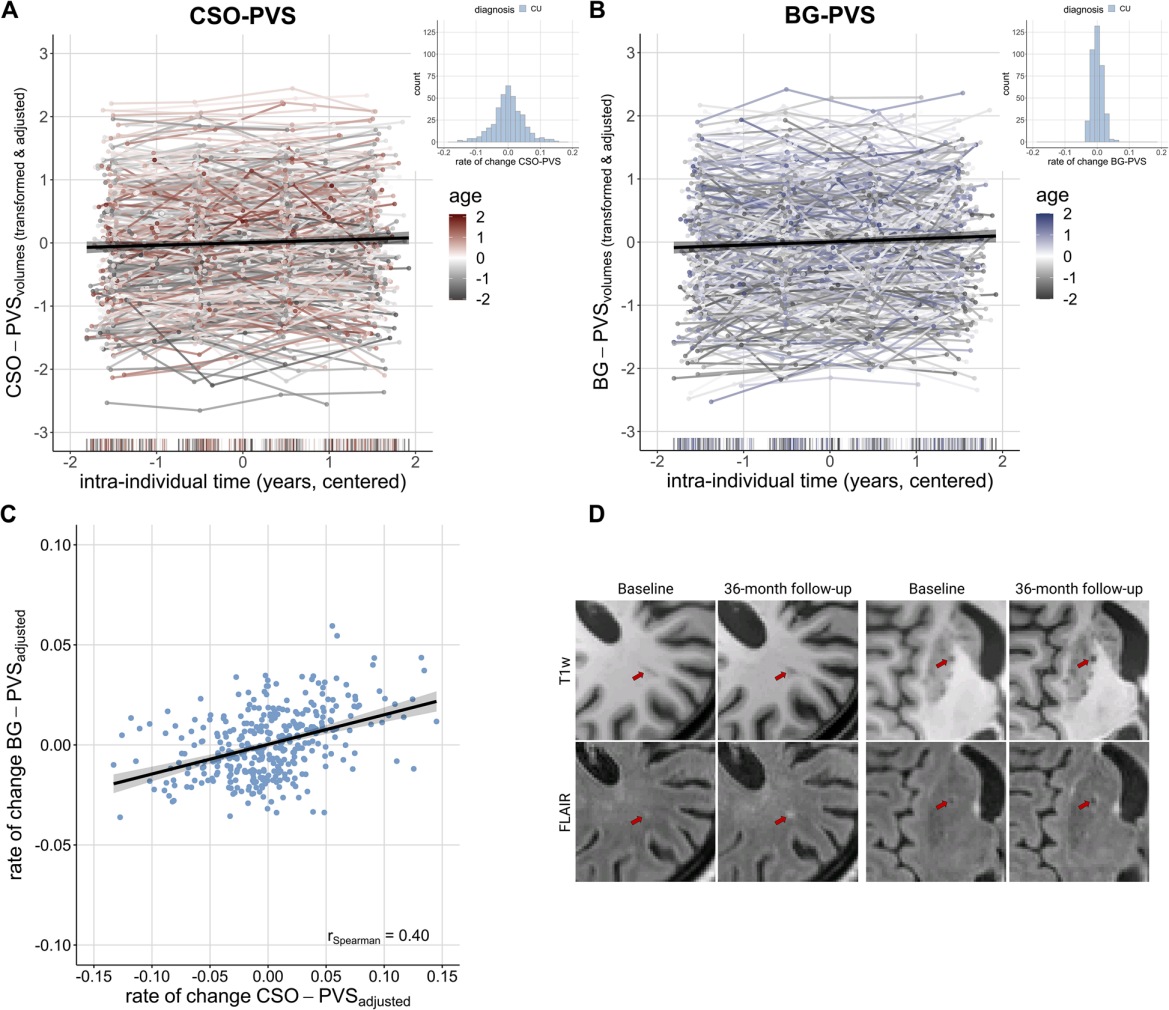
PVS enlargement over follow-ups and with advancing age in cognitively unimpaired subjects. **A**-**B** CSO- and BG-PVS volumes increased over time (CSO: *B* = 0.03 [*95%-CI* 0.02, 0.05], *p* < 0.001; BG: *B* = 0.05, *95%-CI* [0.03, 0.07], *p* < 0.001). Histograms show respective distribution of rates of change in cognitively unimpaired individuals, using transformed data, corrected for effects of age, sex, education, total intracranial volume and regional volumes of WMH of presumed vascular origin. We Box-Cox transformed and z-scored PVS volumes. Plotted PVS volumes were furthermore adjusted for effects of sex, education and total intracranial volume. **C** Moderate, positive correlation of CSO- and BG-PVS change rates. Change rates were corrected for linear and quadratic age effects, sex, years of education, sbTIV as well as regional volumes of WMH of presumed vascular origin. **D** Within a three-year period, volumes of WMH of presumed vascular origin may emerge in the vicinity of PVS. Example images of a CU participant from two different axial slices

PVS volumes generally increased over a three-year period in CU individuals (CSO: *B* = 0.04 [*95%-CI* 0.02, 0.05], *p* < 0.001; BG: *B* = 0.05, *95%-CI* [0.03, 0.07], *p* < 0.001; Fig. 2A, B**; **Table 2). PVS enlargement rates were approximately 0.04 ml/year in CSO and 0.01 ml/year in BG, corresponding to 2.11%/year and 1.82%/year respectively relative to baseline (Table 1). However, the magnitude of this change as well as the baseline volume of PVS varied significantly among individuals, suggesting both increases and decreases. These differences were even slightly more pronounced in CSO compared to BG (random intercept_CSO_ = 0.74, intercept_BG_ = 0.69; random slope_CSO_ = 0.01, random slope_BG_ = 0.00; *see histograms in* Fig. 2A, B).

**Table 2:** Linear mixed-effect modelling for CSO-PVS and BG-PVS in cognitively unimpaired subjects. Models show different trajectories over time, effects of age, sex and years of education. Models with correlated slope and random intercept: PVS ~ age + age^2^ + time + sex + years of education + total intracranial volume + (1 + time | subject)

	**CSO-PVS**				**BG-PVS**			
	***B***	***SE***	***CI***	***p***	***B***	***SE***	***CI***	***p***
(Intercept)	0.03	0.09	-0.14 – 0.20	0.733	0.09	0.09	-0.08 – 0.26	0.307
**age (linear)**	**0.12**	**0.04**	**0.02 – 0.22**	**0.017**	**0.19**	**0.05**	**0.09 – 0.28**	**< 0.001**
**age (quadratic)**	**-0.13**	**0.04**	**-0.22 – -0.04**	**0.006**	**-0.09**	**0.05**	**-0.18 – -0.00**	**0.047**
**time**	**0.04**	**0.01**	**0.02 – 0.05**	**< 0.001**	**0.05**	**0.01**	**0.03 – 0.07**	**< 0.001**
sex	0.18	0.08	-0.07 – 0.43	0.166	0.12	0.12	-0.25 – 0.24	0.959
years of education	-0.01	0.04	-0.11 – 0.08	0.830	-0.01	0.05	-0.10 – 0.08	0.831
**sbTIV**	**0.23**	**0.06**	**0.10 – 0.35**	** < 0.001**	0.09	0.06	-0.03 – 0.21	0.150
σ^2^	0.09				0.18			
τ_00_	0.74 _Subject_				0.69 _Subject_			
τ_11_	0.01 _Subject.time_				0.00 _Subject.time_			
ρ_01_	0.01_Subject_				-0.25_Subject_			
ICC	0.89				0.79			
N	383 _Subject_				387 _Subject_			
Observations	1364				1381			
Marginal R^2^ / Conditional R^2^	0.066 / 0.901				0.058 / 0.804			

Annotations. σ^2^ = residual variance; τ_00_ = random intercept variance; τ_11_ = random slope variance; ρ_01_ = covariance between random slope and intercept

Within the same ROI, baseline PVS volumes weakly associated with PVS change rates: individuals with higher baseline CSO-PVS volumes tended to show higher CSO-PVS change rates (correlation between random intercept and slopes = 0.06). Conversely, those with higher BG-PVS volumes at baseline tended to show less increase in BG-PVS volumes over time (correlation between random intercept and slopes = -0.25). Across ROIs, we observed that individuals with higher CSO-PVS volumes often had higher BG-PVS volumes at baseline (*ρ*_*spearman*_ = 0.57, *p*_*FDR*_ < 0.001). Similarly, those who had higher rates of change in CSO-PVS volumes also had higher rates of change in BG-PVS volumes (*ρ*_*spearman*_ = 0.40, *p*_*FDR*_ < 0.001; Fig. 2C).

#### Predictors of individual PVS differences and change rates

##### Demographics

We next assessed additional predictors of baseline PVS volumes. Chronological age emerged as the primary contributor (CSO: *B* = 0.12, *SE* = 0.05, *95%-CI* [0.02, 0.21], *p* = 0.017; BG: *B* = 0.19, *95%-CI* [0.09, 0.28], *p* < 0.001). However, there was also evidence of a negative quadratic age effect, pointing to deceleration of PVS enlargement in old age (CSO: *B* = -0.13, *SE* = 0.05, *95%-CI* [-0.22, -0.04], *p* = 0.006; BG: *B* = -0.09, *95%-CI* [-0.18, -0.00], *p* = 0.047).

No sex differences or associations with years of education were discernible in PVS volumes in these regions, neither at baseline nor in longitudinal analyses.

##### Hypertension

Baseline PVS volumes (CSO: *W* = 21,551, *p* = 0.136, *r* = 0.09; BG: *W* = 20,479, *p* = 0.575, *r* = 0.030) and PVS rates of change (CSO: *W* = 18,076, *p* = 0.998, *r* < 0.001; BG: *W* = 18,028, *p* = 0.832, *r* = 0.012) did not differ between treated hypertensive and normotensive CU individuals. Additional supplementary analysis on the effect of hypertension by diagnostic groups did not reveal interactive effects of both conditions on PVS volume at baseline or their increase (Supplementary Table 5, Additional File 1).

##### White matter hyperintensities

Individuals with higher baseline PVS volumes tended to have higher baseline volumes of WMH of presumed vascular origin. This showed regardless of whether PVS and WMH of presumed vascular origin were assessed across the same region (CSO: *ρ*_*spearman*_ = 0.10, *p*_*FDR*_ = 0.027; BG: *ρ*_*spearman*_ = 0.21, *p*_*FDR*_ < 0.001) or not (BG-PVS and CSO-WMH: *ρ*_*spearman*_ = 0.14, *p*_*FDR*_ = 0.002; subcortical hyperintensities and CSO-PVS: *ρ*_*spearman*_ = 0.09, *p*_*FDR*_ = 0.051).

Individuals with the highest rates of change in BG-PVS volumes also had the lowest baseline subcortical hyperintensities volume (*ρ*_*spearman*_ = -0.17, *p*_*FDR*_ = 0.001) and CSO-WMH (*ρ*_*spearman*_ = -0.14, *p*_*FDR*_ = 0.006). We did not observe a significant association between CSO-PVS rates of change and baseline CSO-WMH. Visual inspections provided some indication for hyperintensities forming around PVS in various subjects (Fig. 2D).

### Examining PVS dynamics in relation to Alzheimer’s disease

We next studied effects of AD markers on PVS enlargement in the entire sample. We found increasing PVS volumes in the sample of combined diagnostic groups (CSO: *B* = 0.03 [*95%-CI* 0.02, 0.05], *p* < 0.001; BG: *B* = 0.05, *95%-CI* [0.03, 0.07], *p* < 0.001; Supplementary Table 6, Additional File 1).

#### AD syndromal cognitive stages

**Fig. 3:**
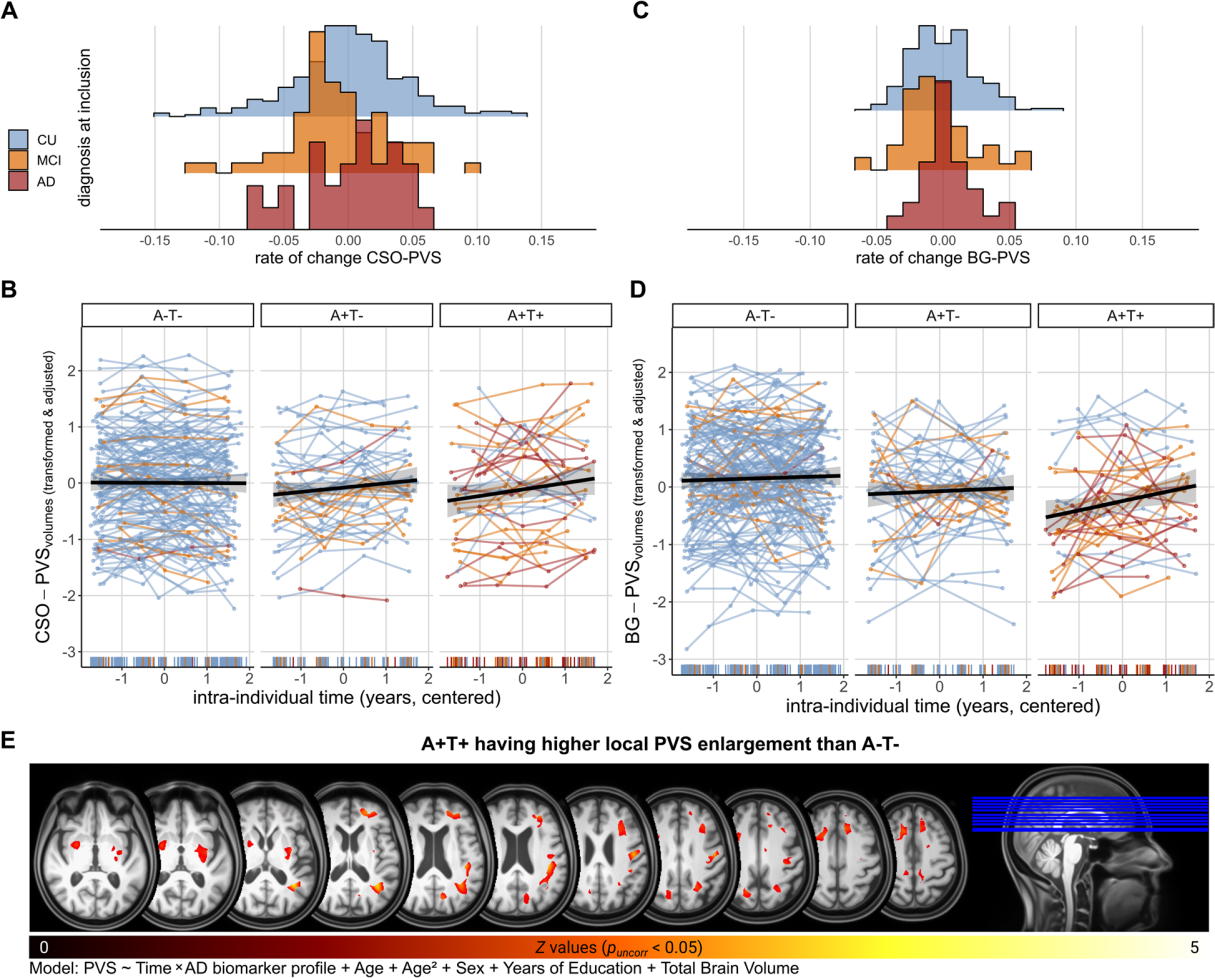
PVS enlargement is associated with AD biomarker profiles. PVS volumes in all plots were Box-Cox and *z*-transformed and adjusted for linear and quadratic age effects, sex, years of education, total intracranial volume and regional WMH of presumed vascular origin. **A** Histogram ridgeline plots of distribution of CSO-PVS rates of change across diagnostic groups. **B** Increase of CSO-PVS is dependent on the AD biomarker profile. Colour of individual trajectories corresponds to diagnosis at time of entry to DELCODE study. Subjects with A + T- or A + T + status show higher rates of change as compared to A-T-. **C** Histogram ridgeline plots of distribution of BG-PVS rates of change across diagnostic groups. **D** Increase of BG-PVS is dependent on the AD biomarker profile. Colour of individual trajectories corresponds to diagnosis at time of entry to DELCODE study. Subjects with A + T + status show higher rates of change as compared to A-T-. **E** Contrast image highlighting regions where PVS enlargement was more evident in A + T + vs. A-T- (*p*_*uncorr*_ < 0.05). We registered all PVS segmentation maps to a DELCODE-specific Multi-Brain (MB) toolbox template [58] and adjusted for local volume changes introduced by normalisation in PVS segmentation maps by modulation with Jacobian determinants [59, 60]. PVS maps were smoothed with Gaussian kernels (6 mm full width at half maximum). Model was aligned with regional marginal models [54] (PVS ~ Time*AT profile + Age + Age.^2^ + Sex + Years of Education + Total Brain Volume)

PVS change rates did not differ across diagnostic groups (CSO: *Χ*^*2*^ (2)= 3.70, *p* = 0.158, *η*^*2*^ = 0.008, Fig. 3A; BG: *Χ*^*2*^(2) = 4.37, *p* = 0.112, *η*^*2*^ = 0.009, Fig. 3C). Similarly, baseline CSO-PVS volumes were comparable across CU, MCI, and AD (*Χ*^*2*^(2) = 0.695, p = 0.706, η^2^ = 0.001). Baseline BG-PVS volumes, on the other hand, varied across diagnostic groups (*Χ*^*2*^(2) = 6.49, *p* = 0.04,* η*^*2*^ = 0.01), with AD showing less BG-PVS volumes than CU (*p*_*FDR*_ = 0.034) and MCI (*p*_*FDR*_ = 0.034). These baseline differences were nonetheless attributable to smaller NABP volumes across these groups (Supplementary Fig. 3 C-F, Additional File 1).

#### AD pathology associates to PVS enlargement

AD biomarker profiles significantly explained inter-individual differences in PVS rates of change (CSO: *Χ*^*2*^(2) = 10.6, *p* = 0.005, *η*^*2*^ = 0.050, Fig. 3B; BG: *Χ*^*2*^(2) = 10.3, *p* = 0.006, *η*^*2*^ = 0.05; Fig. 3D). In the CSO, post-hoc tests revealed that CSO-PVS rates of change were higher in participants with amyloid positivity. Specifically, A-T- participants had lower CSO-PVS rates of change compared to A + T- (*p*_*FDR*_ = 0.021) and A + T + (*p*_*FDR*_ = 0.021), who exhibited an approximate annual PVS enlargement of 0.08 ml/year and 0.14 ml/year respectively (Table 3). However, there was no significant difference between A + T- and A + T + participants. In the BG, post-hoc tests showed that A + T + individuals, exhibiting an approximate annual PVS enlargement of 0.03 ml/year (Table 3), showed significantly faster increase compared to A-T- (*p*_*FDR*_ = 0.004), and a trend compared to A + T- (*p*_*FDR*_ = 0.070). There was no significant difference between A-T- and A + T-.

**Table 3:** Descriptive statistics by AD biomarker profiles for CSO-PVS and BG-PVS volumes (in ml) for four annual visits. Group-level results included subjects who had at least three scans available. We removed extreme values in volumes within the whole sample for each time point to lessen the influence of outliers and corrected estimates for age at the time of the scan

		***AD biomarker profile***		
		**A-T-**	**A+T-**	**A+T+**
*Baseline volume (ml)*	**CSO-PVS**	1.93±0.13	1.63±0.22	1.60±0.25
	**BG-PVS**	0.60±0.03	0.52±0.05	0.44±0.05
*12-month follow-up volume (ml)*	**CSO-PVS**	2.06±0.15	1.81±0.25	1.62±0.29
	**BG-PVS**	0.62±0.03	0.54±0.05	0.51±0.05
*24-month follow-up volume (ml)*	**CSO-PVS**	1.92±0.14	1.89±0.23	1.85±0.26
	**BG-PVS**	0.63±0.03	0.55±0.05	0.50±0.05
*36-month follow-up volume (ml)*	**CSO-PVS**	2.12±0.17	1.87±0.29	1.99±0.36
	**BG-PVS**	0.63±0.03	0.54±0.05	0.54±0.07
*Annual change*^a^ of PVS (in ml/year)	**CSO-PVS**	0.04±0.04	0.08±0.03	0.14±0.03
	**BG-PVS**	0.01±0.00	0.01±0.01	0.03±0.01
*Annual change *^a^ of PVS from baseline (in %/year)	**CSO-PVS**	2.21	4.76	9.03
	**BG-PVS**	1.59	1.26	6.61

Annotations. Mean ± standard error;

^a^Change of PVS volumes per AD biomarker group was estimated based on linear approximations

At baseline, differences between biomarker profiles were not evident in CSO-PVS volumes (*Χ*^*2*^(2) = 2.10, *p* = 0.349, *η*^*2*^ = 0.009) but in BG-PVS volumes (*Χ*^*2*^(2) = 8.81, *p* = 0.01,* η*^*2*^ = 0.04), where A + T + showed lower BG-PVS volumes than A-T- (*p*_*FDR*_ = 0.014). These baseline differences were nonetheless attributable to smaller NABP volumes across these groups (Supplementary Fig. 3 C-F, Additional File 1).

## Discussion

We quantified and monitored PVS volumes across 503 participants from a large German cohort, encompassing various syndromal cognitive stages of AD. Our objective was to characterise longitudinal PVS dynamics and pinpoint potential exacerbating factors. We contributed to current efforts to elucidate the vascular contributions to neurodegeneration from both methodological and medical standpoints [32]. First, we demonstrated that studying PVS enlargement computationally and longitudinally in a large-scale multicentre study is technically feasible and reliable. Second, we provided longitudinal evidence that demonstrated an increase in PVS volumes during ageing and its acceleration with AD pathology (baseline AD biomarker positivity). The stability of longitudinal measurements, along with their meaningful associations with various factors, implies that PVS could be a promising additional biomarker in studying and understanding healthy as well as pathological ageing.

### PVS volumes increase during ageing

The steady rise in PVS volumes observed across follow-ups indicates that ageing is a primary factor driving PVS enlargement, consistent with previous studies [5, 6, 33]. Remarkably, this finding held true whether the longitudinal analyses focused exclusively on participants without objective cognitive impairment or also included those with cognitive impairment. In the presumably healthy ageing sample, we observed an approximate annual increase relative to baseline of 2.11% for CSO-PVS and 1.82% for BG-PVS.

Baseline PVS volumes were generally higher in older participants. However, our analysis also revealed that the extent of this enlargement was limited, as indicated by saturation effects observed in our data and previous work [7]. Though elusive, factors such as the scale of enlargement over short time periods, limitations in imaging resolution (e.g. partial volume effects), and methodological confounds caused by the presence or formation of WMH of presumed vascular origin around PVS and cerebral atrophy, as described in this and previous work [10, 34, 61], may help explain this situation. In addition to group-level increase of PVS, our longitudinal findings revealed substantial unexplained individual differences in change.

We did not observe sex differences in contrast to cross-sectional studies [27, 62].

### PVS and arterial hypertension

Cardiovascular risk factors can promote microvascular alterations and injuries [10], and have been found to be associated with BG-PVS [3, 22, 63]. However, we did not find sufficient evidence for a difference between normotensive and treated hypertensive CU individuals regarding baseline PVS volumes or rates of change. The cohort’s inclusion and exclusion criteria, which effectively render this study one with a comparatively low vascular risk profile, could potentially explain the lack of more evident associations. First, individuals with uncontrolled hypertension were excluded at recruitment. Second, those with a history of hypertension were typically prescribed antihypertensive medication, which has previously been shown to facilitate a reduction in fractional PVS volumes [64].

### PVS and white matter hyperintensities

CU individuals with higher initial volumes of WMH of presumed vascular origin tended to exhibit greater PVS volumes. Interestingly, over time, WMH of presumed vascular origin can form around PVS (as observed in Fig. 1D), emphasising their interdependence and potentially shared underlying cardiovascular or ageing-related mechanisms [22, 34, 65]. Additionally, baseline volumes of WMH of presumed vascular origin and BG-PVS rates of change were correlated with one another, suggesting possible recurrent time-lagged associations. In fact, PVS have been postulated as an early biomarker of cerebral small vessel disease (CSVD) alterations [5, 6], which can precede the development of WMH of presumed vascular origin [34] and accelerate white matter and grey matter deterioration (*for a review, see *[66]). We note, nonetheless, that a focus on change-change modelling, which was not in the scope of this work, is required to further improve our understanding of the chronological ties between dynamics of PVS and WMH of presumed vascular origin. Additionally, the correlation between WMH of presumed vascular origin and PVS progression could also be partially driven by confounding effects of our segmentation method: Since PVS were only segmented in NABP, a faster progression of WMH of presumed vascular origin might have virtually influenced PVS estimation, potentially explaining the absence of associations between baseline WMH of presumed vascular origin with CSO-PVS rates of change and the negative correlation with BG-PVS rates of change.

### PVS and AD pathology

PVS change rates were comparable across diagnostic groups, but varied across AD biomarker profiles, suggesting a closer link to biological factors in this sample. PVS enlargement may represent a structural response to increased neurotoxic waste deposition [12]. In this study, the combined presence of amyloid and tau pathology was most strongly linked to accelerated PVS enlargement, supporting the clearance hypothesis in humans [16, 17, 19]. Strikingly, we also found indications of regional differences; the rate of change in CSO-PVS accelerated with amyloid positivity, whereas it steepened in BG-PVS with combined amyloid and tau positivity. The enlargement of BG-PVS could thus be secondary to brain atrophy, manifesting as ex-vacuo dilatation [67, 68]. Although further research is undoubtedly required, this outcome might suggest that PVS enlargement across these two regions occurs at different stages of pathological ageing (CSO-PVS enlargement in earlier stages and BG-PVS enlargement in more advanced ones, cf. [29]). We note, however, that vicious cycles are likely as well, i.e., neurotoxic waste accumulation can promote clearance pathway failure and vice versa.

Notably, despite having less NABP and consequently less measurable PVS in T1w-based segmentations, participants with AD biomarker positivity (A + T- and A + T +) exhibited higher PVS change rates compared to those with biomarker negativity (A-T-). A comprehensive understanding of PVS dynamics thus requires specialised analyses to explore the dynamic interplay between PVS and other neuroradiological features of CSVD and AD (e.g., via latent change score models). This is particularly true for longer-term longitudinal studies, during which PVS, WMH of presumed vascular origin, and atrophy can undergo significant changes simultaneously.

Moreover, changes in BG-PVS volumes were correlated with baseline volumes of WMH of presumed vascular origin volumes, indicating that these may reflect a combination of cerebrovascular and neurodegenerative processes. As we did not observe additional direct associations with hypertension, our findings lend only partial support to the ongoing discussion about the spatial heterogeneity of PVS aetiology, where BG-PVS enlargement reflects ageing and cardiovascular risk, while CSO-PVS enlargement indicates pathological ageing in the context of AD [19, 22, 25, 63].

Note that AD biomarkers alone accounted for only a small portion of the variability in PVS rates of change. This implies that other conditions, beyond those considered in this work, also contribute to PVS changes. Lifestyle factors [69] as well as genetics [70] have been associated with the presence and abundance of PVS in the brain and could therefore be conceivable contributors to PVS dynamics. (Chronic) neuroinflammation might also contribute to PVS enlargement [42, 71] and potentially explain why PVS change rates are concurrently associated with CSVD and AD. The accumulation of peripheral immune cells and cytokines in the PVS could impair glymphatic clearance [2, 42, 72], ultimately leading to PVS enlargement. Neuroinflammation could be triggered in response to, prior to, and during blood–brain barrier dysfunction [73–75] and by the accumulation of neurotoxic waste and the formation of amyloid plaques [76, 77]. We therefore propose that PVS dynamics are at the crossroads of multiple influential factors, and we encourage future modelling studies to investigate the joint impact of multiple factors on PVS dynamics.

### Limitations and open questions

PVS segmentation leveraged T1w and FLAIR imaging, deviating from the recommended T2w and FLAIR imaging assessment [46]. We emphasize that this decision was made after careful evaluation of the pros and cons of using either partial head coverage or anisotropic full head coverage T2w imaging. We refrained from using partial head coverage T2w imaging due to the exclusion of frontal and occipital brain areas, and inconsistent head coverage over time, which would have compromised PVS measurement reliability. We also refrained from using anisotropic full head coverage T2w imaging since it was only available for a small subset of participants (*n* = 214 at baseline) and since anisotropy could compromise PVS assessments [49]. Clearly, there was a cost involved in the choice of T1w-based PVS segmentation; PVS could only reasonably be assessed in NABP, making this study more prone to underestimating PVS. Nonetheless, we opted for this approximation to increase the study power while still enabling valid interpretation. In fact, we found that T2w-based and T1w-based PVS segmentations agreed similarly with clinical visual ratings.

We emphasise that, for the time being, computerised segmentation provides a surrogate estimate of PVS burden and that clinical assessment and validation remain essential. Moreover, automatic segmentation of PVS allow for the investigation of additional morphological features (e.g. PVS width, length [8, 48, 78]) which would enable determining whether PVS enlargement corresponds e.g. to a widening of the already-visible PVS or an increase in PVS counts. Both phenomena may be intertwined and could have distinct clinical implications.

We additionally identified five open questions. First, we noted that demographics accounted for only a small portion of the variability in PVS volumes. Factors not included in this study, e.g. genetics [70], lifestyle [69], or neuroinflammation [42, 71] might explain variability in baseline and longitudinal PVS measurements. Second, we narrowed the scope of our work to linear dynamics of PVS progression. We encourage further longitudinal investigations to precisely examine their temporal dynamics. Third, trends of increased PVS burden across AD biomarker stages suggest that PVS might serve as an additional indicator for cognitive decline. Findings in this area are still conflicting [4, 79] and require further careful investigation. Fourth, PVS dynamics do not seem disconnected from other pathological processes, making it sensible to examine their interactions over time to better understand disease pathogenesis and progression and identify clinical phenotypes that can help in deriving potential interventions. Fifth, although our study provides evidence of an association between neurotoxic waste accumulation and PVS enlargement, we refrain from asserting direct cause-consequence relationships. Given the limited availability of CSF-derived AD biomarkers, we additionally advise careful interpretation. Moreover, it is well conceivable that PVS enlargement—at least in parts—may be a dynamic, adaptive reaction to e.g. neurotoxic waste accumulation or neuroinflammation (cf. PVS in multiple sclerosis [80]). Disentangling temporary, adaptive from chronic, mal-adaptive PVS enlargement is a challenge for future studies.

## Conclusion

Collectively, our work suggests that ageing is a primary driver of PVS enlargement and that cycles of neurotoxic waste accumulation may also contribute to this process. Our findings contribute to the understanding of PVS progression as a biomarker in the context of neurodegenerative diseases. PVS might be an early marker of AD pathophysiological cascades and through comprehensive understanding of their pathological dynamics they might have the potential to facilitate the development of early interventions. Further research is required to disentangle pathological cascades, their concurrent dynamics, and their unique contributions to disease progression.

## Supplementary Information

Additional file 1: Supplementary Figure 1. Data availability flowchart across all four measurement time points. Supplementary Figure 2. Exemplary comparison between T1w and T2w partial head coverage imaging in the full sample. Supplementary Figure 3. Diagnostic group differences in normal appearing brain parenchyma (NABP) and volumes of WMH of presumed vascular orgin across ROIs. Supplementary Figure 4: Flow chart of the main steps of the PVS segmentation pipeline to assess perivascular spaces morphometrics. Supplementary Figure 5: Correlation plots comparing regional PVS measurements obtained via pooled and multi-site optimisation. Supplementary Figure 6: PVS segmentation using a subsample with T2w scans. Supplementary Table 1: Information on scanner models used on the 503 subjects at baseline. Supplementary Table 2: Information on head coils used on the 503 subjects at baseline. Supplementary Table 3: Segmentation thresholds obtained using pooled and multi-site optimisation. Supplementary Table 4: Multiple linear regression relating baseline CSO-PVS and BG-PVS volumes to age (linear and quadratic), sex, years of education, and sbTIV. Supplementary Table 5: ANOVA results on interaction effects of hypertension and diagnostic group on CSO-PVS and BG-PVS volumes at baseline and their rate of change. Supplementary Table 6: Linear mixed effect modelling for CSO-PVS and BG-PVS in entire sample, showing different trajectories over time, effects of age, sex and years of education.

Additional file 2.

## Data Availability

The data and materials that support this study are not publicly available, but may be provided upon reasonable request.

## Abbreviations

Aβ: Amyloid-β
AD: Alzheimer’s disease
BG: Basal ganglia
CCC: Lin's concordance correlation coefficient
CERAD-plus: Consortium to Establish a Registry for Alzheimer’s Disease
CSF: Cerebrospinal fluid
CSO: Centrum semiovale
CSVD: Cerebral small vessel disease
CU: Cognitively unimpaired
DELCODE: DZNE Longitudinal Cognitive Impairment and Dementia Study
LME: Linear mixed-effects
MCI: Mild cognitive impairment
MRI: Magnetic resonance imaging
NABP: Normal-appearing brain parenchyma
pTau181: Phosphorylated tau181
PVS: Perivascular spaces
ROI: Region-of-Interest
sbTIV: Segmentation-based total intracranial volume
WMH: White matter hyperintensities
