# Supplementary material for: Perivascular space enlargement accelerates in ageing and Alzheimer’s disease pathology: evidence from a three-year longitudinal multicentre study
Source: Alzheimers Res Ther. 2024 Oct 31;16:242. doi: 10.1186/s13195-024-01603-8 (PMC11526621; doi:10.1186/s13195-024-01603-8)
Supplement: Supplementary file 2 — Additional file 2. [file 13195_2024_1603_MOESM2_ESM.pdf]

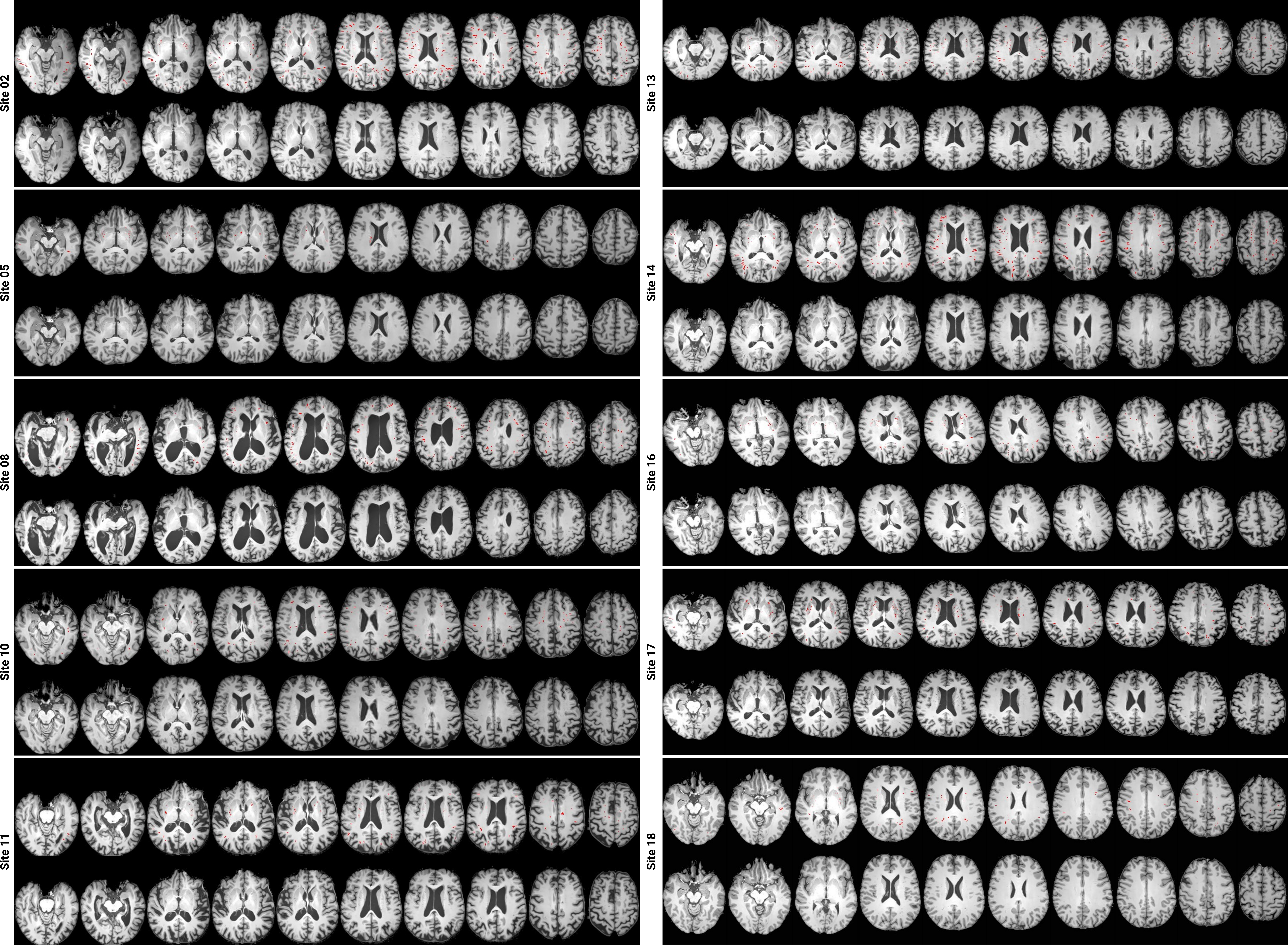

Site 02
Site 05
Site 08
Site 10
Site 11
Site 13
Site 14
Site 16
Site 17
Site 18
