# Supplementary material for: Perivascular space enlargement accelerates in ageing and Alzheimer’s disease pathology: evidence from a three-year longitudinal multicentre study
Source: Alzheimers Res Ther. 2024 Oct 31;16:242. doi: 10.1186/s13195-024-01603-8 (PMC11526621; doi:10.1186/s13195-024-01603-8)
Supplement: Supplementary file 1 — Additional file 1: Supplementary Figure 1. Data availability flowchart across all four measurement time points. Supplementary Figure 2. Exemplary comparison between T1w and T2w partial head coverage imaging in the full sample. Supplementary Figure 3. Diagnostic group differences in normal appearing brain parenchyma (NABP) and volumes of WMH of presumed vascular orgin across ROIs. Supplementary Figure 4: Flow chart of the main steps of the PVS segmentation pipeline to assess perivascular spaces morphometrics. Supplementary Figure 5: Correlation plots comparing regional PVS measurements obtained via pooled and multi-site optimisation. Supplementary Figure 6: PVS segmentation using a subsample with T2w scans. Supplementary Table 1: Information on scanner models used on the 503 subjects at baseline. Supplementary Table 2: Information on head coils used on the 503 subjects at baseline. Supplementary Table 3: Segmentation thresholds obtained using pooled and multi-site optimisation. Supplementary Table 4: Multiple linear regression relating baseline CSO-PVS and BG-PVS volumes to age (linear and quadratic), sex, years of education, and sbTIV. Supplementary Table 5: ANOVA results on interaction effects of hypertension and diagnostic group on CSO-PVS and BG-PVS volumes at baseline and their rate of change. Supplementary Table 6: Linear mixed effect modelling for CSO-PVS and BG-PVS in entire sample, showing different trajectories over time, effects of age, sex and years of education. [file 13195_2024_1603_MOESM1_ESM.pdf]

Additional File 1

Supplementary Material

2 Methods

2.1 Study design and participants

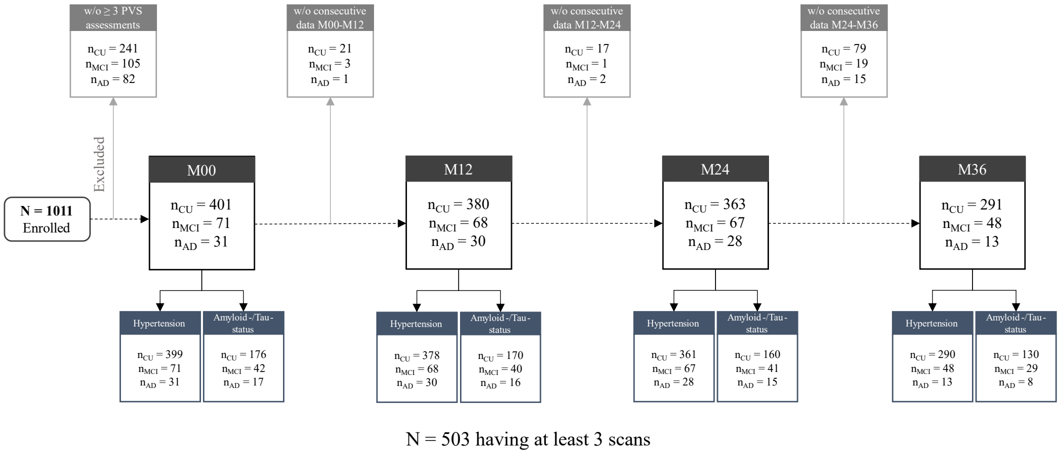

Supplementary Figure 1. Data availability flowchart across all four measurement time points.

2.1.3 Structural magnetic resonance imaging

Approximately 96% of the participants in this study were scanned using Siemens Skyra, TrioTim, or Verio scanners (n=119, 204, and 162, respectively; Supplementary Table 1). All sites used Siemens coils, which included 12, 20, 32, or 64-channel head coils. The 12 and 20-channel coils were used for participants with larger heads who could not fit in the 32-channel and 64-channel coils. About 80% of the participants were imaged using the 32-channel head coil (Supplementary Table 2).

Supplementary Table 1. Information on scanner models used on the 503 subjects at baseline.

Model	Prisma	Prisma fit	Skyra	Skyra fit	TrioTim	Verio	Total
n	15	1	119	2	204	162	503

Supplementary Table 2. Information on head coils used on the 503 subjects at baseline.

Model	12 channels	20 channels	32 channels	64 channels	Total
n	3	78	421	78	503

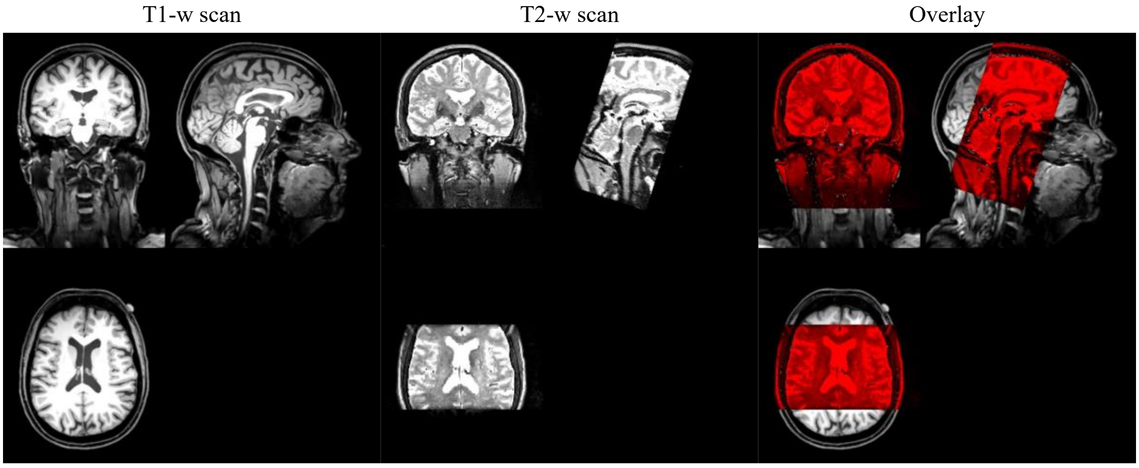

Supplementary Figure 2. Exemplary comparison between T1w and T2w partial head coverage imaging in the full sample. T2w partial head coverage images were anisotropic (0.5 × 0.5 × 1.5 mm, 384 × 384 px, 64 quasi-coronal slices perpendicular to hippocampal long axis, TR/TE 3500/353 ms), and did not cover the entire brain, limiting the availability, reliability and quality of PVS quantification. Full head T2w imaging (not depicted here), was anisotropic, too (0.8 × 0.8 × 2 mm, 240 × 320 px, 72 axial slices, TR/TE 6500/79 ms) and only available in a subsample of n=214. We hence opted for a T1w and FLAIR assessment of PVS.

2.2 Segmentation and quantification

2.2.1 Total intracranial volume

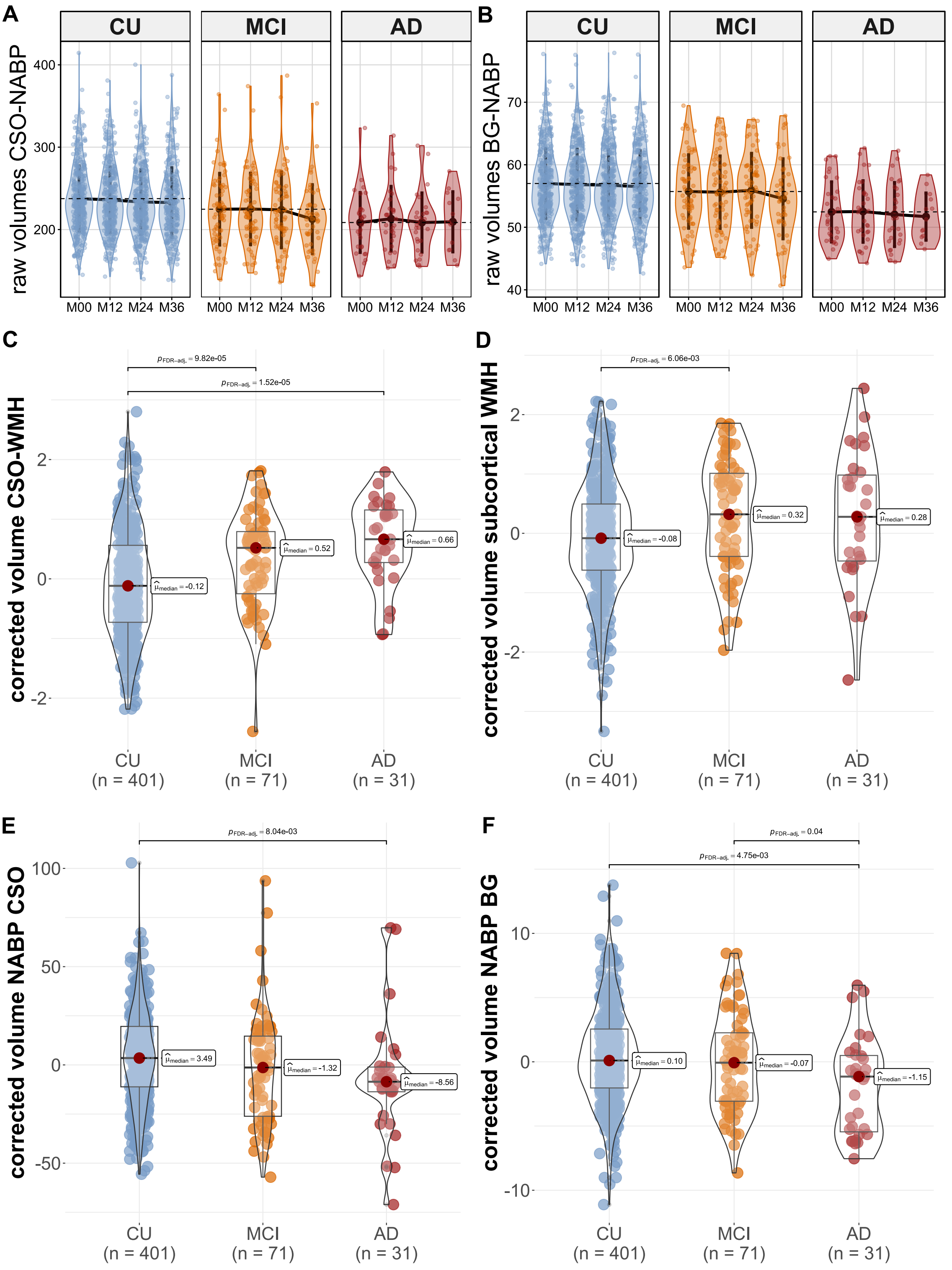

Supplementary Figure 3. Diagnostic group differences in normal appearing brain parenchyma (NABP) and volumes of WMH of presumed vascular origin across ROIs. (A-B) We here show-case differences and decreases of NABP volumes within in ROIs across the diagnostic groups.

Changes in NABP would render the commonly used measures fractional PVS volumes confounded over time, as an increase in fractional PVS volumes may correspond to an increase in PVS volumes per se or a shrinkage of the ROI. We hence opted to use the total intracranial volume (sbTIV) as a covariate since this measurement remains stable over time. (C-D) Using non-parametric testing, we investigated differences in baseline volumes of WMH of presumed vascular origin (corrected for effects of age, sex, years of education and sbTIV) across diagnostic groups. Volumes of WMH of presumed vascular origin differed across diagnostic groups (CSO-WMH: $X^2(2) = 33.3$, $p < 0.001$, $\eta^2 = 0.063$; subcortical WMH: $X^2(2) = 11.8$, $p = 0.003$, $\eta^2 = 0.020$), with CU having lower volumes than MCI (CSO-WMH: pFDR< 0.001; subcortical WMH: pFDR= 0.006) and AD (CSO-WMH: pFDR< 0.001; subcortical WMH: pFDR= 0.100). (E-F) Using non-parametric testing, we investigated differences in volumes of NABP at baseline (corrected for effects of age, sex, years of education and sbTIV) across diagnostic groups. NABP volumes varied across diagnostic groups (CSO: $X^2(2) = 11.7$, $p = 0.003$, $\eta^2 = 0.019$; BG: $X^2(2) = 10.4$, $p = 0.005$, $\eta^2 = 0.017$), with AD having smaller NABP volumes than CU (CSO: pFDR = 0.008; BG: pFDR= 0.005). Moreover, AD exhibited smaller BG-NABP than MCI (pFDR= 0.045).

2.2.2 Segmentation and parameter tuning

We ran the FreeSurfer recon-all command on T1w images to obtain white matter parcellations and aggregated these parcellations to create BG, CSO, and brainstem masks. We further refined these masks to guarantee the exclusion of the ventricular atrium, choroid plexus, and posterior horns of the lateral ventricles—areas prone to mis-segmentation with FreeSurfer—via brain atlas registration(1). We segmented PVS based on T1w images using the Frangi filter and optimised thresholding(2,3). We used connected component analysis to filter out too large or too small PVS candidates. We eliminated structures that were positioned completely at white matter perimeter in order to mitigate partial volume effects(4,5). We segmented white matter hyperintensities (WMH) using a hierarchical thresholding approach leveraging T1w and FLAIR imaging to restrict our PVS measurements to the normal-appearing brain parenchyma (NABP). We estimated total PVS volumes within CSO and BG.

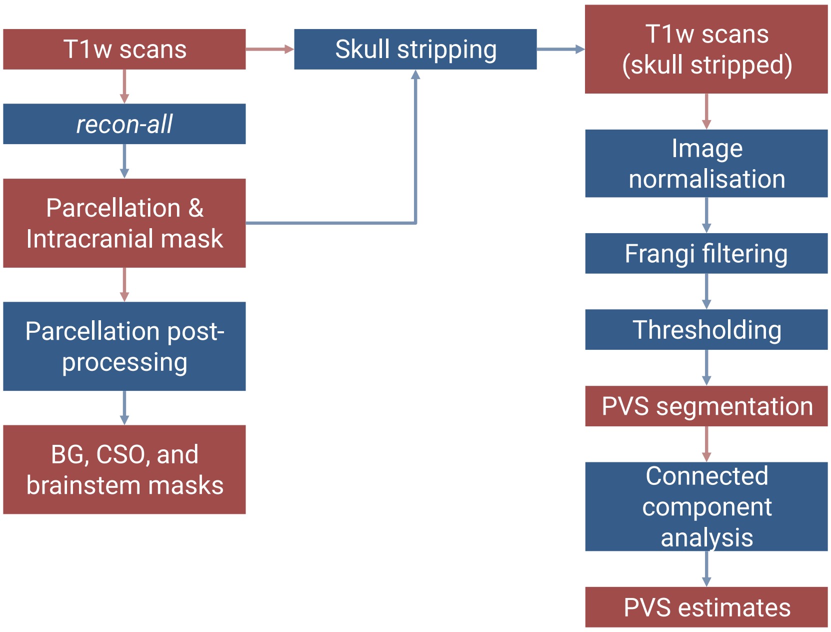

Supplementary Figure 4. Flow chart of the main steps of the PVS segmentation pipeline to assess perivascular spaces morphometrics.

2.2.2.1 Segmentation and parameter tuning

PVS optimisation

We compared the segmentation results with optimisation accounting for data from different sites versus when it did not. Throughout the following discussion, we refer to these approximations as multi-site and pooled optimisation, respectively. In both cases, optimisation involved finding segmentation thresholds per ROI that maximized the Spearman correlation between visual PVS ratings (6) and computational PVS volumes, similar to the work of (3). The main difference between the two experiments was the number of parameters optimised—either 10 (multi-site) or 1 (pooled) per ROI. The segmentation thresholds for each ROI and each optimisation strategy are condensed in Supplementary Table 3. We compared the corresponding sets of PVS measurements with each other, with clinical visual ratings, and with demographic variables.

Supplementary Table 3. Segmentation thresholds obtained using pooled and multi-site optimisation. We present results independently for each region of interest (ROI).

ROI	Pooled optimisation	Multi-site optimisation
**BG**	1.6854 × 10-5	1.6823 [95%-CI 1.6441, 1.7204] × 10^{-5}
**CSO**	2.5074×10-6	2.5717 [95%-CI 2.3801, 2.7633] × 10^{-6}

We first used Spearman correlation to evaluate the similarity between computational PVS volumes obtained via multi-site and pooled optimisation approach. Both sets of

measurements were strongly correlated with one another (Supplementary Figure 5 C-D; CSO: $\rho_{spearman}$ = 0.89, p < 0.001; BG: $\rho_{spearman}$ = 0.94, p < 0.001).

We then used polyserial correlation to assess the agreement between clinical PVS ratings and PVS volumes obtained via pooled and multi-site optimisation (Figure 1 A-B and Supplementary Figure 5 A-B). Both sets of measurements resulted in moderate and comparable polyserial correlations.

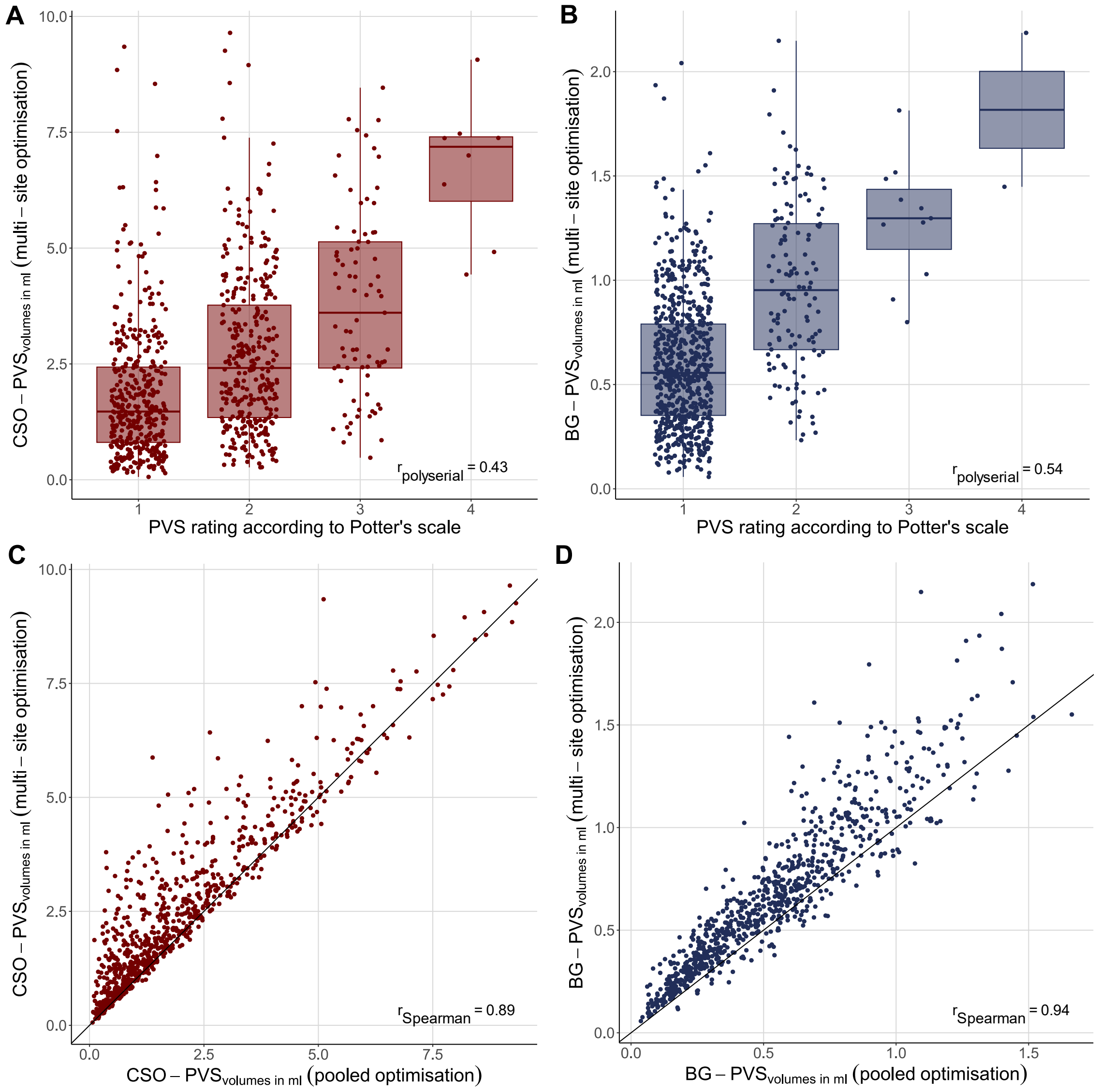

Supplementary Figure 5. Multi-site optimisation approach for PVS segmentation. (A-B) Relationship between visual scores and PVS volumes derived from a multi-site optimisation strategy. (B-C) Correlation plots comparing regional PVS measurements obtained via pooled and multi-site optimisation. The diagonal grey solid line represents the identity function, and as such, most data points were expected to cluster around this line.

Lastly, we examined baseline PVS measurements obtained through each optimisation approach in relation to baseline demographic data. We used multiple linear regression

for this analysis. The models assessed associations between PVS volumes (Box-Cox transformed) as the outcome and independent predictors, namely age (both linear and quadratic terms), sex, years of education, and sbTIV. We used separate models for each region of interest and each optimisation approach. We noticed that both the sign and magnitude of the beta coefficients were similar between the models using PVS measurements obtained through either optimisation approach (Supplementary Table 4).

Supplementary Table 4. Multiple linear regression relating baseline CSO-PVS and BG-PVS volumes to age (linear and quadratic), sex, years of education, and sbTIV. Computational PVS volumes were acquired using pooled and multi-site optimisation.

Predictor	**Dependent variable**							
	BG volumes				CSO volumes			
	Pooled		Multisite		Pooled		Multisite	
	B (SE)	P	B (SE)	P	B (SE)	P	B (SE)	P
**Intercept**	0.04 (0.06)	0.421	0.04 (0.05)	0.415	0.13 (0.06)	0.020	0.13 (0.06)	0.020
**Age (linear)**	0.21 (0.04)	<0.001	0.21 (0.04)	<0.001	0.08 (0.04)	0.056	0.08 (0.04)	0.063
**Age (quadratic)**	-0.04 (0.04)	0.230	-0.04 (0.04)	0.224	-0.13 (0.04)	0.001	-0.13 (0.04)	0.001
**Sex**	-0.06 (0.06)	0.266	-0.06 (0.06)	0.292	0.13 (0.06)	0.019	0.14 (0.06)	0.013
**Years of education**	-0.02 (0.04)	0.589	-0.03 (0.04)	0.462	-0.00 (0.04)	0.934	-0.00 (0.04)	0.982
**sbTIV**	0.07 (0.06)	0.215	0.09 (0.06)	0.100	0.19 (0.06)	0.001	0.20 (0.06)	<0.001
**R2**	0.065		0.068		0.041		0.042	

Across all of these assessments, both optimisation strategies yielded comparable results. To simplify the process, we opted for a pooled optimisation. We would like to highlight that pooled optimisation may not always yield results comparable to those from a multi-site approach, especially when protocols differ substantially across sites. In all instances, we recommend using an assessment approach similar to the one employed here to determine which optimisation strategy is most appropriate.

References

1. Dickie DA, Job DE, Rodriguez D, Robson A, Danso S, Pernet C, et al. Brain Imaging of Normal Subjects (BRAINS) Age-Specific MRI Atlases from Young Adults to the Very Elderly (v1.0), [dataset]. University of Edinburgh, Edinburgh Imaging, CCBS, BRAINS Imagebank. 2016;

2. Frangi AF, Niessen WJ, Vincken KL, Viergever MA. Multiscale vessel enhancement filtering. In: Lecture Notes in Computer Science. 1998. p. 130–7.

3. Ballerini L, Lovreglio R, Valdés Hernández MDC, Ramirez J, MacIntosh BJ, Black SE, et al. Perivascular Spaces Segmentation in Brain MRI Using Optimal 3D Filtering. Sci Rep. 2018;8(1):1–11.

4. Bernal J, Valdés-hernández MC, Escudero J, Duarte R, Ballerini L, Mark E. Assessment of PVS enhancement methods using a three-dimensional computational model. 2022;(April).

5. del C. Valdés Hernández M, Piper RJ, Wang X, Deary IJ, Wardlaw JM. Towards the automatic computational assessment of enlarged perivascular spaces on brain magnetic resonance images: A systematic review. Journal of Magnetic Resonance Imaging. 2013 Oct;38(4):774–85.

6. Potter GM, Chappell FM, Morris Z, Wardlaw JM. Cerebral perivascular spaces visible on magnetic resonance imaging: Development of a qualitative rating scale and its observer reliability. Cerebrovascular Diseases. 2015;39(3–4):224–31.

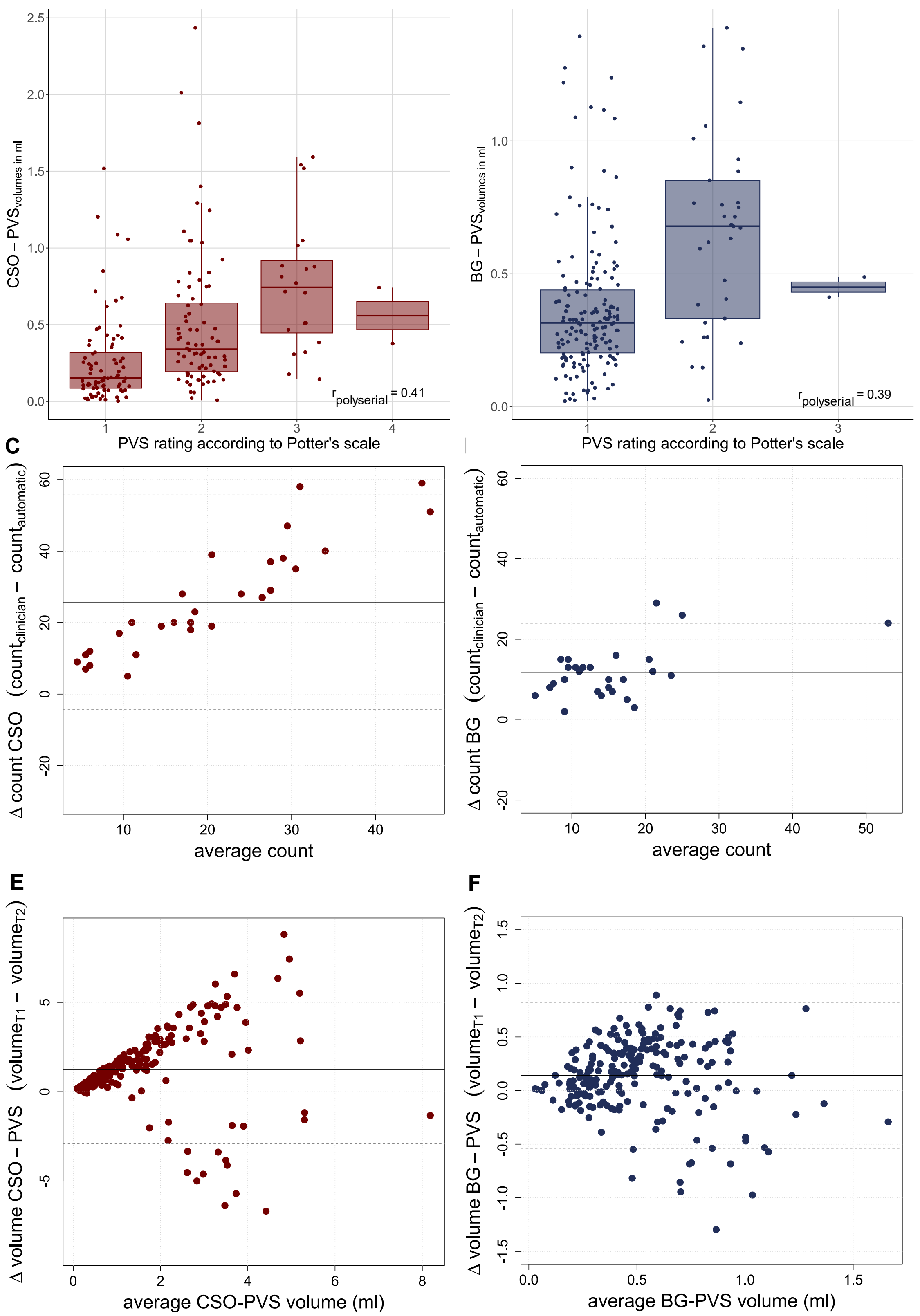

Supplementary Figure 6. PVS segmentation using a subsample with T2w scans. For this validation part, we used the subsample with available T2w full head coverage imaging (n=214). (A-B) We observed moderate polyserial correlations between computational PVS volumes and visual scores assessed from T2w scans with partial head coverage (CSO: $r_{polyserial}$= 0.41, p < 0.001; BG: $r_{polyserial}$= 0.39, p < 0.001). (C-D) In the 30 randomly selected participants, Lin's concordance between visual and computational counts in the same slice were low to moderate

and showed underestimation of computational counts in both regions (CSO: difference$_{mean}$ = 25.72, difference$_{SD}$ = 15.30, CCC = 0.14, 95%-CI [0.06, 0.21]; BG: difference$_{mean}$ = 11.69, difference$_{SD}$ = 6.26, CCC = 0.44, 95%-CI [0.27, 0.59]). Using Spearman's correlation, we observed strong associations between manual counts and computational volumes (CSO: $\rho_{spearman}$ = 0.60, p = 0.001; BG: $\rho_{spearman}$ = 0.68, p < 0.001). (E-F) The direct comparison between PVS volumes assessed from T1w imaging vs from T2w imaging revealed low to moderate Lin's concordance for both regions (CSO: CCC = 0.16, 95%-CI [0.06, 0.26]; BG: CCC = 0.37, 95%-CI [0.26, 0.47]). Compared to PVS volumes assessed from T1w, T2w PVS volumes were generally underestimated, with stronger underestimation in the CSO (difference$_{mean}$ = 1.25, difference$_{SD}$ = 2.12) than in the BG (difference$_{mean}$ = 0.14, difference$_{SD}$ = 0.35). Using Spearman's correlation, we observed strong associations between PVS volumes segmented from T1w and T2w imaging (CSO: $\rho_{spearman}$ = 0.60, p < 0.001; BG: $\rho_{spearman}$ = 0.44, p < 0.001).

3 Results

3.3 PVS enlargement in CU individuals

3.3.1 Predictors of individual PVS differences and change rates

3.3.1.2 Hypertension

In a subsequent supplementary analysis, we also examined interaction effects of the risk factors hypertension (normotensive vs. treated hypertensive) and diagnostic groups (CU vs. MCI vs. AD) on PVS volumes at baseline and their rates of change via 2×3 ANOVAs. We identified outliers (Q3 + 1.5×IQR or below Q1 - 1.5×IQR of group median) and removed them prior to ANOVA.

Supplementary Table 5. ANOVA results on interaction effects of hypertension and diagnostic group on CSO-PVS and BG-PVS volumes at baseline and their rate of change.

			**F (df)**	**p**	***η²***
**baseline**	**CSO**	hypertension	1.76 (1,492)	0.186	0.004
		diagnosis	0.48 (2,492)	0.622	0.002
		hypertension x diagnosis	0.45 (2,492)	0.641	0.002
	**BG**	hypertension	0.13 (1,494)	0.717	0.0002
		diagnosis	3.53 (2,494)	0.030	0.014
		hypertension x diagnosis	0.09 (2,494)	0.917	0.0004
**Rates of change**	**CSO**	hypertension	0.30 (1,447)	0.585	0.001
		diagnosis	0.73 (2,447)	0.482	0.003
		hypertension x diagnosis	2.11 (2,447)	0.122	0.009
	**BG**	hypertension	0.02 (1,465)	0.896	<0.001
		diagnosis	2.04 (2,465)	0.131	0.009
		hypertension x diagnosis	0.70 (2,465)	0.499	0.003

3.4 Examining PVS dynamics in relation to AD

Supplementary Table 6. Linear mixed effect modelling for CSO-PVS and BG-PVS in entire sample, showing different trajectories over time, effects of age, sex and years of education. Models with correlated slope and random intercept: PVS ~ age + age² + time + sex + years of education + total intracranial volume + (1+ time | subject).

	CSO-PVS				BG-PVS			
Predictors	B	SE	CI	p	B	SE	CI	p
(Intercept)	0.06	0.08	-0.09 – 0.22	0.404	0.14	0.08	-0.01 – 0.28	0.074
age (linear)	0.12	0.04	0.04 – 0.21	0.004	0.19	0.04	0.11 – 0.27	<0.001
age (quadratic)	-0.14	0.04	-0.21 – -0.06	<0.001	-0.11	0.04	-0.18 – -0.03	<0.001
time	0.03	0.01	0.02 – 0.05	<0.001	0.05	0.01	0.03 – 0.07	<0.001
Years of education	-0.02	0.04	-0.10 – 0.06	0.572	-0.02	0.04	-0.10 – 0.06	0.638
sex	0.12	0.11	-0.10 – 0.34	0.272	-0.06	0.11	-0.27 – 0-16	0.609
sbTIV	0.19	0.06	0.08 – 0.30	0.001	0.05	0.05	-0.05 – 0.16	0.320
σ2	0.09				0.18			
τ00	0.73 Subject				0.67 Subject			
τ11	0.01 Subject.time_ind				0.00 Subject.time_ind			
ρ01	0.06 Subject				-0.20 Subject			
ICC	0.89				0.79			
N	478 Subject				482 Subject			
Observations	1694				1712			
Marginal R2 / Conditional R2	0.061 / 0.899				0.060 / 0.802			

Annotations. σ^2 = residual variance; τ_{00} = random intercept variance; τ_{11} = random slope variance; ρ_{01} = covariance between random slope and intercept.
